# Terahertz Magneto-Optic Sensor/Imager

**DOI:** 10.1038/s41598-020-58085-5

**Published:** 2020-01-24

**Authors:** Dmitry S. Bulgarevich, Yusuke Akamine, Miezel Talara, Valynn Mag-usara, Hideaki Kitahara, Hiroyuki Kato, Masaki Shiihara, Masahiko Tani, Makoto Watanabe

**Affiliations:** 1 Research Center for Structural Materials, National Institute for Materials Science, 1-2-1 Sengen, Tsukuba, Ibaraki 305-0047 Japan; 2 Research Center for Development of Far-Infrared Region, University of Fukui, Fukui, 3-9-1 Bunkyo, 910-8507 Japan

**Keywords:** Imaging techniques, Electronic and spintronic devices, Magneto-optics, Terahertz optics

## Abstract

We are reporting a new type of compact magneto-optic sensor constructed from terahertz-wave spintronic emitter and electro-optic detector. The corresponding terahertz polarization output of the emitter and the detection phase-sensitivity of the detector depend on the vector of the external magnetic field. The emitter/detector pair consists of two small and thin wafers sandwiched together and capped with a thin gold mirror. As a result, the use of bulky terahertz steering/collection optics was completely eliminated in our magneto-optic imager. With such simple on-chip generation/detection scheme for terahertz time-domain setup in reflection-type geometry, we were able to record the raster-scanned image contrast of a permanent magnet in the proximity of the sensor surface. The contrast strongly varies with the magnet orientation and its position with respect to the sensor. The imager spatial resolution depends on chip optical quality for tight femtosecond-laser pump/probe cross-focusing at detector/mirror interface and terahertz generation/detection efficiency. In this respect, the chip robustness to the pump/probe fluences is also an important factor to consider.

## Introduction

The main advantage of typical magneto-optic imager/imaging (MOI)^1–7^ over magnetic field imager/imaging (MFI) is the possibility of achieving higher spatial resolution by using visible light sources and smaller sizes of the sensing domains. The highest reported spatial resolutions with MOI by using large-area Faraday rotator sensors were between 0.5 and 100 µm, which were limited by the magnetic domain periodicity and thickness of the sensor layer^8–10^. A maximum of 10-µT magnetic field resolution/sensitivity with MOI was observed^8^. Although magneto-optical Kerr effect microscopy (MOKE) can also provide up to 0.2-µm spatial resolution, it is limited to materials with strong Kerr response^11^. The μT range magnetic sensitivity of nitrogen-vacancy centres in diamond can also be used for imaging of stray magnetic fields in thin ferromagnetic films with 0.44-µm spatial resolution^12^.

On the other hand, the MFI with superconducting quantum interference device (SQUID), giant magnetoresistance (GMR), anisotropic magnetoresistance (AMR), extraordinary magnetoresistance (EMR), giant magnetoimpedance (GMI), tunnel magnetoresistance (TMR), Hall effect, microelectromechanical systems (MEMS), etc. sensors can also offer excellent magnetic field sensitivity/resolution up to aT range, but with reduced spatial resolution (worse than 100 µm).

In this respect, the development of new type MOIs for non-destructive testing (NDT) applications with detection of magnetic flux leakage (MFL) above the sample surface is still an important topic in terms of finding the balance between MOI spatial and axial resolutions, magnetic field sensitivity, physical size, robustness, operation conditions, cost, applications, etc. factors. In this work, we describe our first attempt to develop the novel MOI based on recently reported spintronic terahertz (THz) emitters^13–15^ and well-known electro-optic (EO) THz detectors^16^ for THz time-domain spectroscopy (TDS)^17,18^. The magnetic field is the key factor for generation of spintronic THz emission and there are several recent reports on magnetic-field tailoring of the terahertz polarization and amplitude^19–21^. The possibility of external magnetic field distribution mapping using terahertz emission from semiconductor surfaces was also demonstrated^22^. Although, the THz MOI was not reported yet.

## Experimental

**Figure 1:**
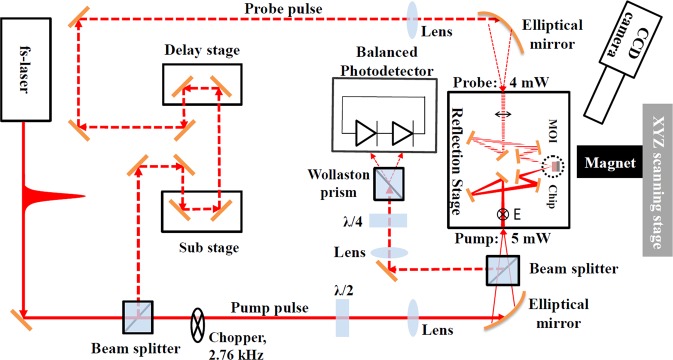
The schematic of the reflection-type THz-TDS MOI setup with EO sampling.

**Figure 2:**
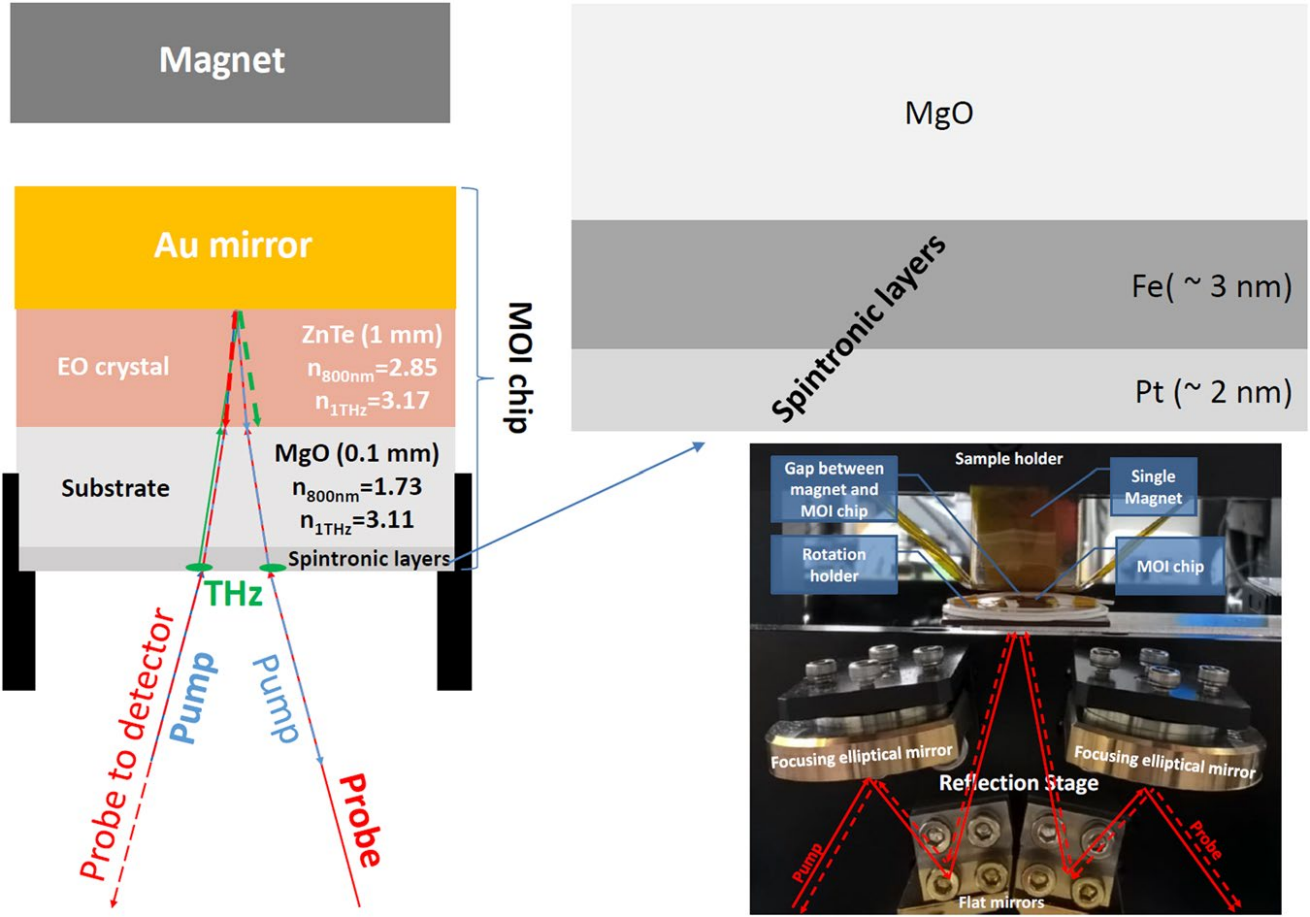
The schematic drawings of the on-chip sensor for THz-TDS MOI, the structure of the THz wave spintronic emitter, and close-up photo with focusing optics, sensor rotation holder, and permanent magnet on XYZ-scanning stage holder.

Figure 1 shows the schematics of the reflection-type THz-TDS imaging setup (avpIRS-500-SP, Aispec Instruments) we used with some modifications to accommodate the EO sampling and achieve tight refocusing for THz generation/detection with our MOI chip^23^. Figure 2 depicts the side view of the MOI chip assembly and the spintronic emitter structure as well as the magnified image of the focusing optics, chip rotation holder, and scanning sample holder with attached permanent magnet. The ~10-fs pump/probe beams (λ = 780 nm central wavelength, 80 MHz repetition rate, 5/4 mW power) emitted from the Integral Element PRO 400 (Spectra-Physics) laser source are incident to the reflection stage with refocusing/steering optics from opposite directions and are cross-focused at the ZnTe/Au-mirror interface. Since good cross-focusing is crucial for THz signal detection, the design allows for the Au-mirror to be removable when a CCD camera with long working distance objective is used for initial alignments/focusing at ZnTe/air interface.

As shown in Fig. 2, the pump beam hits the MOI chip from the spintronic layer side and generates the electron currents in vertical direction within the thin metallic films. If the ferromagnetic (FM) Fe layer is magnetized, the mobility of the majority spin-up electrons is higher compared to that of the minority spin-down electrons. In the non-magnetic (NM) Pt layer with strong spin–orbit interaction, the spin-up and spin-down electrons deflect in opposite directions and produce the ultrafast transverse charge current by inverse spin-Hall effect^24^ (ISHE). This current is the source of the THz radiation from the spintronic layer^13^. The linear polarization of such THz emission is orthogonal to the magnetization vector, it can be modulated/changed even by relatively weak magnetic field (below 10 mT), and it is independent on pump polarization.

Then, THz radiation from the spintronic layer propagates through the MgO substrate and EO ZnTe wafers, reflects from the Au-mirror, and co-propagates inside the ZnTe wafer with the probe beam reflected from the Au-mirror. The divergence angle between the THz/probe fronts (see bold green and red dashed arrows in Fig. 2) is ~15° with our re-focusing optics. At this stage, the probe beam linear polarization is perturbed by the THz electric field in the EO crystal. Finally, the induced ellipticity component of probe polarization is analysed with standard EO-sampling assembly in the THz-TDS setup, which results in the recording of THz waveforms or/and images. Note that initial pump/probe linear polarizations were orthogonal to each other and in-plane with the spintronic layer/air interface. The MOI chip was also aligned in a calibrated rotation holder to have the pump beam polarization orthogonal to the < 110 > axis of ZnTe crystal in order to supress the parasitic THz emission generated inside the EO crystal by optical rectification (OR) due to moderate ~0.2 mJ/cm^2^ pump/probe fluences with our cross-focusing setup.

In this work, we utilized the previously optimized Fe/Pt spintronic bilayer structure for maximum THz emission output^14^. It consists of 2-nm Fe and 3-nm Pt layers (see Fig. 2), which can be grown by molecular beam epitaxy or electron beam evaporation on 500-μm MgO substrate (Tateho Chemical Industries Co., Ltd.). The both side polished ZnTe (110) crystal with 11×9×1 mm^3^ dimensions (Techno Chemics, Inc.) was used for EO detection.

The magnetic field properties of used permanent magnet were visualized and characterized with Magnetic Field Visualize Sheet (C-Task Un-Digital) and Gauss Meter with Hall sensor (Model 410, Lake Shore Cryotronics, Inc), respectively. The image post-processing was done with FIJI software package^25^.

## Results and discussion

**Figure 3:**
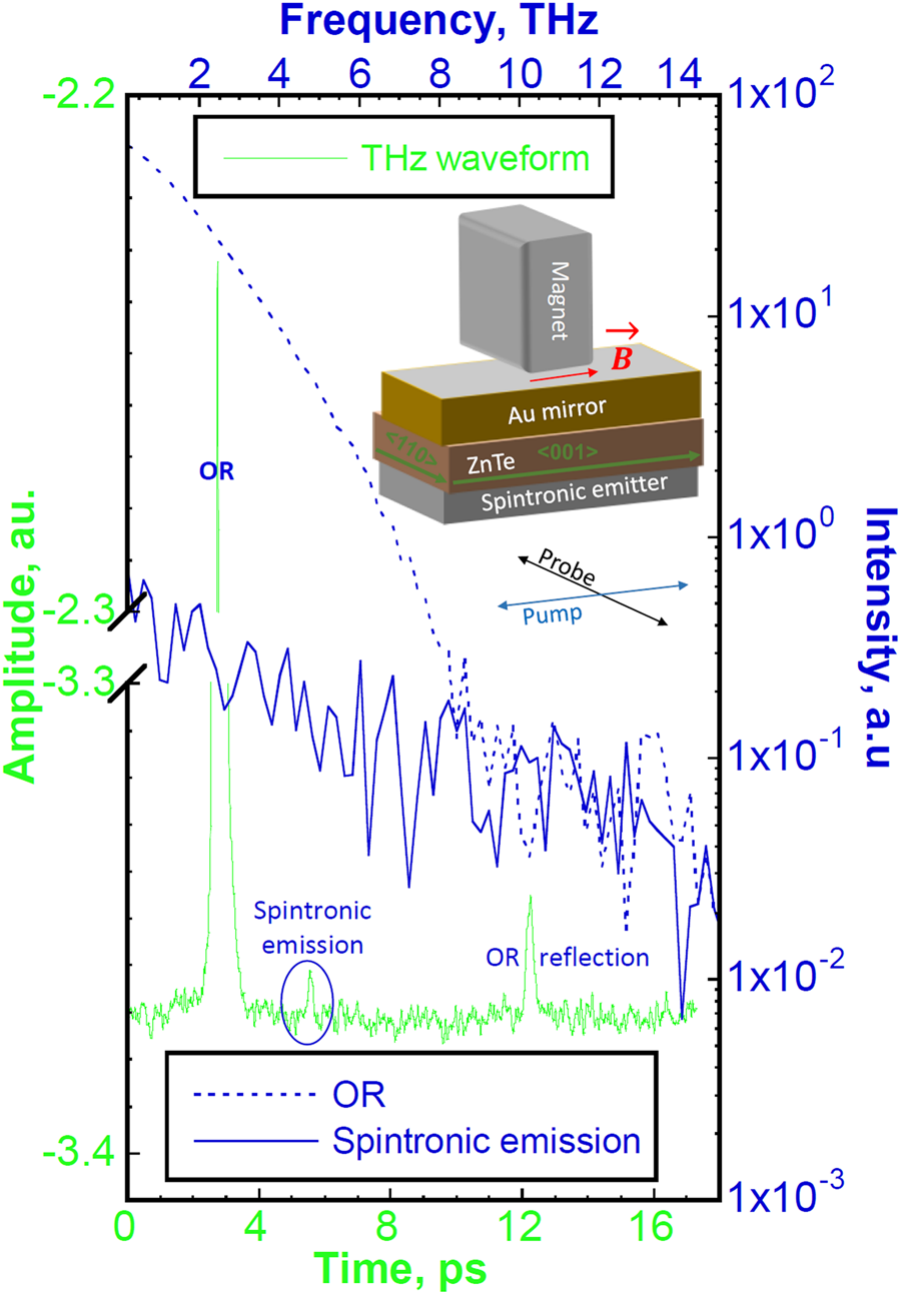
The THz waveform collected with displayed optical setup of MOI chip, permanent magnet, and pump/probe polarizations. The scale colours correspond to the plot ones. For OR and spintronic emissions in frequency domains, the 0–5 and 5–10 ps waveform portions were used in FFT calculations, respectively.

Figure 3 shows the typical unipolar waveform collected by using the indicated magneto-optical setup. One of the advantages of using an on-chip THz generation/detection scheme is that the atmospheric water vapour does not influence the measured signals. For this on-chip THz generation/detection scheme, the main THz peak is insensitive to the magnet position and originates from the parasitic OR of the pump beam in the ZnTe crystal. The weakest signal is delayed from such OR at ~+2.8 ps after propagation to ZnTe/Au interface. Such delay is due to the difference in refractive indexes for optical pump and spintronic-emitter-generated THz fronts in MgO and ZnTe wafers (see Fig. 2). This signal is well separated from the OR peak to get any parasitic effect on the MOI measurements. At constant pump/probe powers, its peak amplitude and phase are affected only by the strength and vector of the external magnetic field (discussed below). After magnet removal, this signal could be registered for days before complete disappearance due to slow de-magnetization of the Fe layer.

The moderate waveform peak at ~+9.5 ps is due to the reflection of the OR-generated THz front at the ZnTe/MgO interface and partial co-propagation in ZnTe with the incoming probe beam (see Fig. 2). As such, this waveform peak is also insensitive to the magnetic field. With currently available pump/probe powers in our MOI setup, the signal-to noise ratio (SNR ≤ 1) is low for spintronic emission, so multiple waveform or signal averaging was used to improve it. With 32 times averaging, the SNR = *A*_*signal*_/*RMS*_*noise*_ ≅ 4, where *A*_*signal*_ is the amplitude of the magnetic field sensitive signal and *RMS*_*noise*_ is the root mean square of the noise amplitude (see Fig. 3 for THz waveform). By using the fast Fourier transform (FFT) on collected waveform, the response in frequency domain for OR and spintronic emissions are also shown for comparison in Fig. 3. With 32 times averaging, the spintronic emission bandwidth is ~4–5 THz.

**Figure 4:**
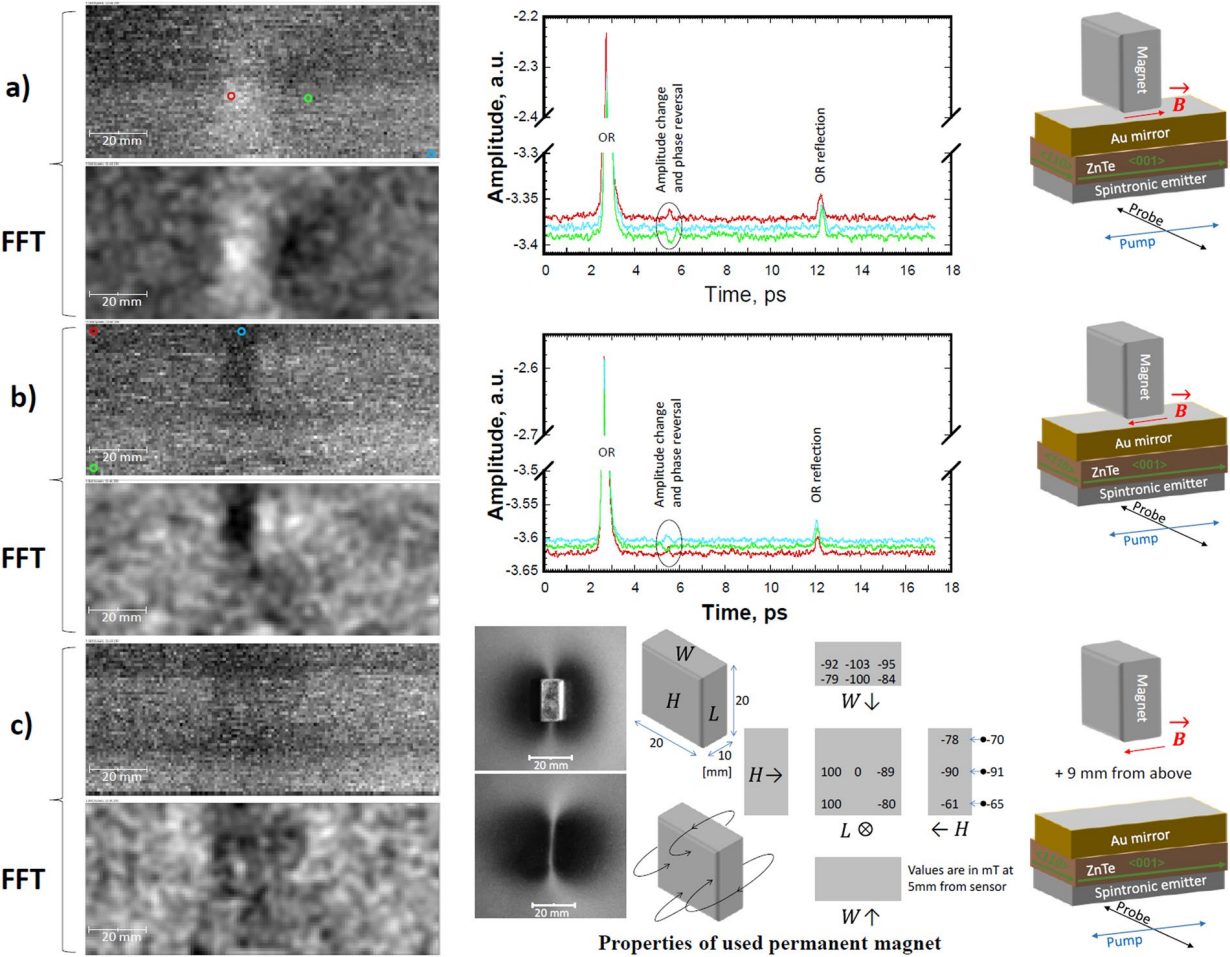
MOI results with different permanent magnet orientation with respect to the on-chip THz sensor (see text for more details). The magnet in (**a**) and (**b**) was ~5 mm above the spintronic layer surface. The waveform colours correspond to ones of the circles, which indicate the spatial positions on images for their collections. The photos of used magnet positioned above and below of the MFVS are in (**c**) together with other drawings and data for magnet properties.

**Figure 5:**
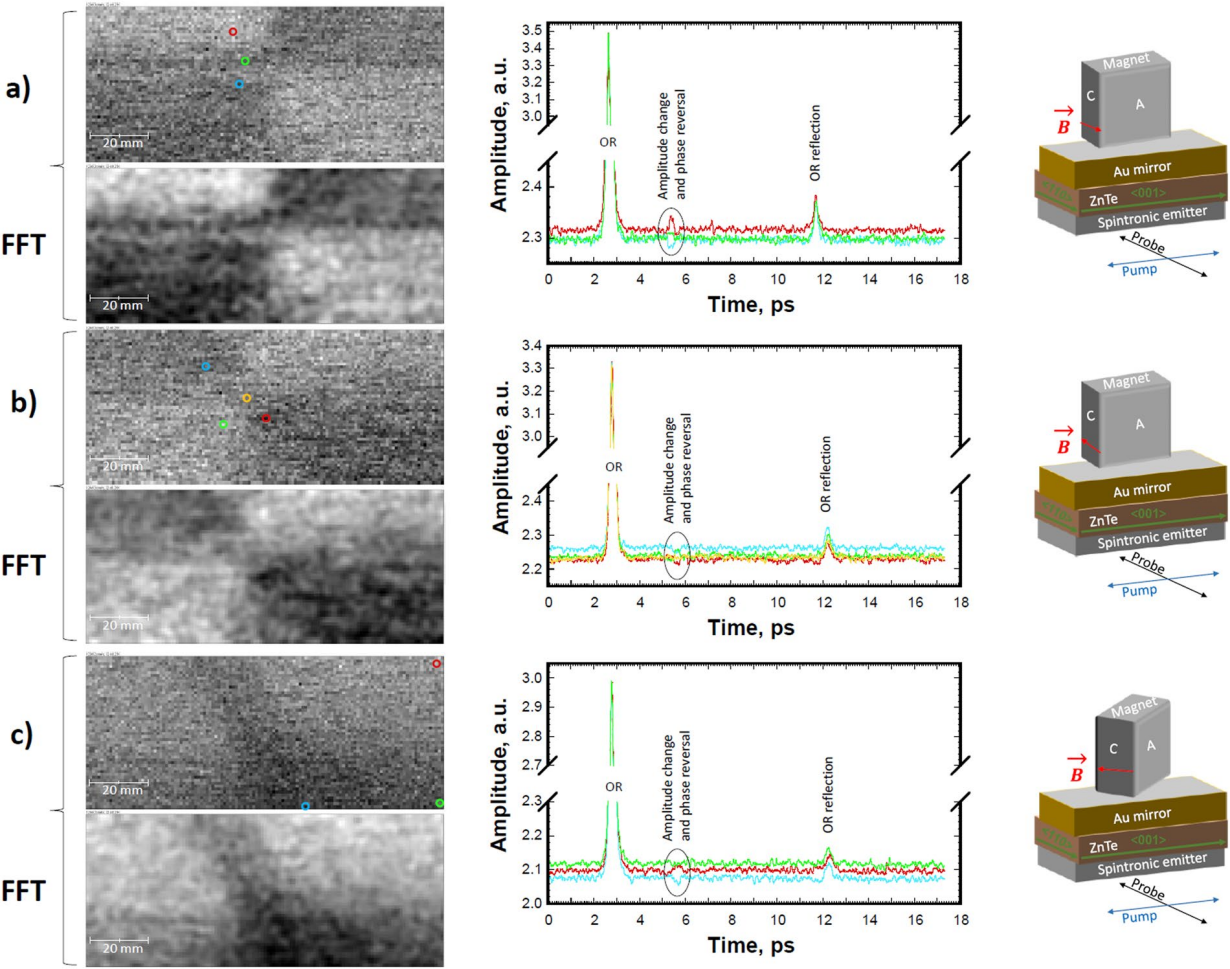
MOI results with different permanent magnet orientation with respect to the on-chip THz sensor. The magnet in (**a**), (**b**), and (**c**) was ~5 mm above the spintronic layer surface. The waveform colours correspond to ones of the circles, which indicate the spatial positions on images for their collections.

Figures 4(a–c) and 5(a–c) show the images for indicated magnetic field vector (*B*) directions in the magnet/MOI-chip setup and oscillating electric field vectors/polarizations (E) of pump/probe beams. They were collected at fixed delay stage position, which corresponded to the magnetic-field-sensitive waveform maximum at ~+2.8 ps from the OR peak maximum. During continuous ~0.5 s/pixel X-scanning stage movement with attached magnet, the waveform amplitude at this delay stage position was sampled 16 times for averaging within each 1×1 mm pixel. It took ~1 h for the collection of each original image shown in Figs. 4 and 5. To improve the image contrast, bandpass filtering with FFT was applied. As shown in Figs. 4 and 5, filtering suppresses the horizontal stripes generated by some THz-TDS system instability. It also smoothens the variations of each image’s bright and dark patches with sizes larger than 40 pixels and strongly attenuates the objects smaller than 3 pixels^26^.

The 32 time-averaged waveforms collected at different spatial positions are also shown in Figs. 4 and 5. These waveforms demonstrate that the signal for magnetic-field-sensitive peak can change not only its intensity depending on MOI-chip vs magnet position/orientation, but also its phase. This explains the image contrast reversal depending on the direction of the magnetic field vector (compare Fig. 4(a,b) as well as Fig. 5(a,b)).

If α is the angle of the THz beam polarization with respect to the (110) axis of ZnTe crystal, then the EO sampling signal will depend on it by a factor of $\sqrt{1+3co{s}^{2}\alpha }$. It should be noted that the polarization of THz emission from the spintronic layer is always orthogonal to the component of the *B* vector oriented in-plane with our MOI sensor surface. This in-plane *B* vector component changes with the magnet position and orientation with respect to the pump beam spot on the MOI sensor during imaging due to the re-magnetization of the spintronic layer at this spot. As a result, the EO sampling detects amplitude and phase variations in the THz signal effected by such re-magnetization, which leads to the possibility of MOI with our sensor chip. In other words, our MOI constantly detects the current magnetization state within the spot of the focused pump beam on the spintronic layer. The resulted MOI is a convolution of spintronic emitter efficiency and EO detector sensitivity to spintronic layer magnetization.

Comparing of THz MOI images with photos of Magnetic Field Visualize Sheet (MFVS) in Fig. 4, it can be seen that THz MOI gives better magnet shape visualization. So far however, the THz MOI has lower sensitivity/resolution to the magnetic field. Note that in Fig. 4 (c) with photos of permanent magnet on top and under of the MFVS, the bright and dark areas indicate the predominance of in-plane and out-of-plane components of the magnetic field lines with respect to the plane of the MFVS surface, respectively. Such magnetic field induced contrast is due to the different reflectivity of aligned nickel flakes (parallel or edge-on to the MFVS surface) suspended in oil micro-capsules between flexible translucent and substrate thin sheets. For areas far away from magnet (not shown), the image contrast indicates the magnetization state history of MFVS unrelated to the current magnet position in Fig. 4 (c). Therefore, our THz MOI demonstrates the consistent results with MFVS.

In principle, the lateral spatial resolution of our MOI should be governed by the optical resolution of the pump/probe focusing optics. The axial resolution depends on the magnetic field sensitivity with EO sampling and can affect the spatial one. This was the case with our current THz-TDS setup. The lateral resolution was low due to poor image contrast brought by a low magnetic field sensitivity. To improve the SNR, a higher pump power is needed. In the current set-up, we used a 5mW (~0.2 mJ/cm^2^ fluence) pump power. Previous study reported that this spintronic layer could withstand up to ~25 times higher fluence^14^. However, the linear relation was observed between pump power and THz output amplitude (slope is ~1) only up to ~0.25 mJ/cm^2^ fluence^15^. Nevertheless, the EO SNR is expected to be improved at higher fluences. For example, it was reported that in nonlinear regime from ~0.25 to ~1.25 mJ/cm^2^, the THz signal amplitude increased ~2 times^27^. Note that 1.25 mJ/cm^2^ fluence is still ~5 times lower than our spintronic layer damage threshold.

In addition, we expect that by modifying the spintronic layer structure into the antenna shape, the THz output amplitude and beam directivity could improve the EO signal at least 8 times^28^. Further investigations on spintronic layer materials with large spin Hall angle^29^, layer thickness, and layer sequence could lead to additional improvements. The spintronic layer composition also could be optimized to have the wider/smoother dependence of THz emission amplitude on magnetic field strength^21^. Recently, the sub-mT sensitivity of THz emission was reported with Co_20_Fe_60_B_20_/Pt spintronic bilayer^30^.

Moreover, the optical losses could be decreased by eliminating the use of MgO and glass substrates and directly microfabricating the spintronic and mirror layers on EO-detector chip surface. Besides EO sampling, the development of suitable on-chip photoconductive antenna or spintronic detector is another way to improve the THz MOI sensitivity. It is also expected that magnetic field modulation will increase the SNR. As for image collection speed, the use of higher pump/probe modulation rate could also enhance the image scanning velocity. In summary, we expect that SNR with our MOI could be improved with additional studies/modifications.

## Concluding remarks

To our best knowledge, we are reporting the first MOI with on-chip emitter/detector sensor for THz-TDS. By improving its SNR, this technique could significantly contribute to the very limited number of reported MOI schemes for NDT applications. Potentially, our scheme has an advantage over Faraday-based and Kerr effect-based techniques in terms of likely achievable spatial resolution and applicability to various materials.
